# Extraordinary diversity of the pinniped lactation triad: lactation and growth strategies of seals, sea lions, fur seals, and walruses

**DOI:** 10.1093/af/vfad037

**Published:** 2023-06-14

**Authors:** Julie P Avery, Steven A Zinn

**Affiliations:** Water and Environmental Research Center, Institute of Northern Engineering, University of Alaska, Fairbanks, AK 99775, USA; Department of Animal Science, University of Connecticut, Storrs, CT 06269, USA

**Keywords:** length of lactation, marine mammals, milk composition, nursing behavior, pup growth rate

**Implications**

- Marine mammals produce a high fat (>25%‐60%), energy rich milk that facilitates rapid growth of offspring.
- Maternal provisioning in pinnipeds, varies from 4 days (hooded seal) to 24 months (Pacific walrus).
- Marine mammals evolved different lactation strategies, including extended periods of fasting in the mother and the offspring influenced by environment yet tightly associated with phylogeny.
- A mutation in alpha-lactalbumin gene is present in otariids (sea lions and fur seals) that prevents lactose production and is thought to allow for maintenance of lactation even with long (up to 23 days) inter-suckling interval by preventing mammary involution.
- Growth strategies of pinnipeds are partially influenced by milk composition (high percentage of fat) and rate of energy delivery (total calories provided); however, offspring physiology (metabolic hormones) and behavior (increased activity) also impact the rate and composition of mass gain which strongly predict offspring survival.

## Introduction

Mammalian reproductive success is highly influenced by early postnatal survival due to the high mortality in the neonatal and post-weaning (transition to independent foraging) periods. Lactation is an essential and critical life history event with significant implications for offspring survival and hence female reproductive success (Lee, 1996). Lactation is influenced by intrinsic and extrinsic factors from dam and offspring; however, recent research has begun to consider milk as the third component of the lactation triad encouraging additional research of milk as an integrated biological system (Christian et al., 2021).

Marine mammals have exceptional diversity in nursing strategy thought to have evolved to maximize offspring success for unique environments (Schulz and Bowen, 2004). These maternal lactation strategies are closely tied to off-spring growth strategies. This review will examine the extreme and diverse lactation and growth strategies of pinnipeds including true seals, sea lions, fur seals, and walrus. This review, while not exhaustive, provides an overview of unique adaptations of the pinniped lactation triad with select species examples.

## Marine Mammal Overview

Marine mammals have developed unique lactation strategies that facilitate energy transfer in a fully aquatic or semiaquatic environment. Marine mammals are distributed among four Orders of mammals: Mysticeti (baleen whales), Odontoceti (toothed whales, dolphins, and porpoise), Sirenia (manatee and dugong), and Carnivora [polar bears (Ursidae), sea otters (Mustlidae), walrus (Odobenidae), true seals (Phocidae), sea lions and fur seals (Otariidae)]. These species have an exceptional diversity of lactation strategies that vary by maternal energy acquisition and delivery [capital (fasting) vs. income (foraging) investment strategy], duration of lactation, and composition of milk that result in differential relative and absolute growth rates of pups as well as composition of body mass gain.

Given the unique logistical challenges of studying lactation in a large, fully aquatic organism, this review will focus primarily on semiaquatic pinnipeds where more detailed studies of lactation physiology and pup growth have been completed. Pinnipeds, including walrus, seals, sea lions, and fur seals, are considered semiaquatic because they are tied to the land or ice for key life history events such as lactation, breeding, and molting; however, aquatic foraging is required for energy acquisition and repletion.

Length of lactation in pinnipeds varies from the shortest lactation of any mammal, 4 days in arctic hooded seals (*Cystophora cristata*), to 18 months in the temperate to subtropical Australian sea lion (*Neophoca cinerea*), and 2‐3 years in the subarctic walrus (*Odobenus rosmarus*) (Fay, 1982) although no consistent relationship has been observed between latitude and milk composition (Schulz and Bowen, 2004). Comparison of lactation among diverse species is challenging given differences in duration. While most mammals are still providing colostrum in the first 3‐5 days, hooded seals have delivered 10 L of high fat milk per day (Oftedal et al., 1993).

## Behavioral Ecology—Lactation Strategy

Terrestrial mammals have two primary lactation strategies that can be termed “cash” or “carry”. Species like small rodents utilize a cash strategy where young are left in a nest for short periods while females forage for food, and females return to the nest to provide nutrients to the offspring. In the carry strategy, offspring is always in close proximity to the female and are nursed on demand. This carry strategy is utilized by primates, including humans and hoofstock.

Three distinct lactation strategies are observed in marine mammals: aquatic lactation, capital-fasting, and income-foraging strategies. The aquatic lactation strategy utilized by walrus, sirenians, and dolphins is the most similar to the terrestrial “carry” strategy where the offspring remain in close proximity to the cow with nursing on demand that is initiated by the offspring. This provisioning strategy often lasts for multiple years and affords the opportunity for significant maternal investment for lactation and social learning (Noren et al., 2014; Mann, 2019; Sepúlveda and Harcourt, 2021). Walruses are the only pinniped species that exhibit this strategy; nursing on demand for more than 2 years (Schulz and Bowen, 2004). Walruses are shallow, benthic foragers; thus, limited diving and foraging abilities of young walrus pups likely do not limit female acquisition of prey facilitating this aquatic lactation strategy (Fay, 1982; Noren et al., 2014).

**Figure 1.**
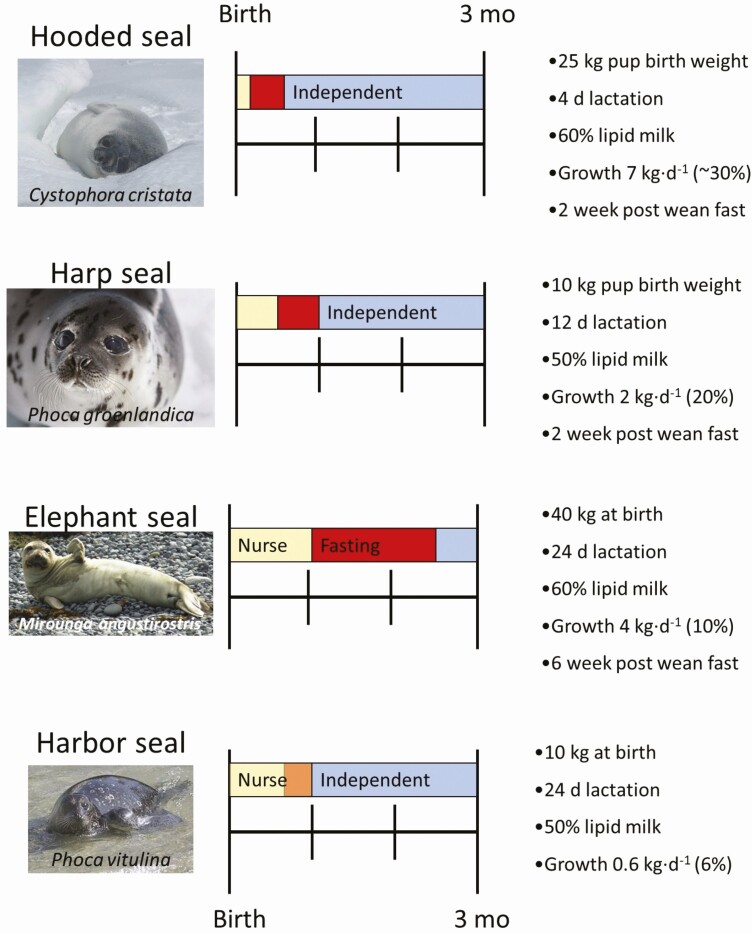
Graphical representation of the Capital-Fasting Maternal Investment Strategy utilized by Phocids (true seal) in four different species. Species common name above image with genus species in italic within image. At birth, pups are nursed persistently (yellow) from 4 to 24 days, while the dam is fasting. Pups are abruptly weaned followed by a 2‐6 week post-weaning fast (red) where dams are no longer attending pups and pups are not consuming other food resources. Duration of fast is dependent on species and acquired pup energy reserves stored as blubber (subcutaneous adipose). Small bodied harbor seals (bottom panel) do not have the energy reserves for a strict post-weaning fast and instead weaning pups gradually (orange) with supplementation of small forage fish and extended maternal care where foraging behavior is learned. Harbor seal strategy is more similar to the otariid (sea lion and fur seal) Investment-Foraging Strategy. True independent foraging (blue) includes only fish consumption. Variation in provisioning strategy results in differential growth rates from 6% to 10% of birth weight gained per day during nursing (Schulz and Bowen, 2004).

Most true seals (phocids) and all baleen whales (mysticetes) exhibit a capital-fasting lactation strategy (Figure 1) where all energy capital to be provided to offspring is acquired before parturition at distant foraging grounds (Schulz and Bowen, 2004; Irvine et al., 2017). During energy demanding lactation, females do not eat to replenish energy provisioned to offspring. Other than bears, mysticetes and phocids are the only other mammalian species where lactation and forging are temporally and spatially separated (Oftedal, 1993). Females fast for weeks (phocids) to months (mysticetes). Large maternal body size relative to offspring is key to this strategy, and females must have predictable, high quality foraging grounds to acquire enough energy before parturition to successfully transfer all needed energy, primarily as fat, to their offspring (Oftedal, 1993; Irvine et al., 2017).

The capital-fasting strategy appears to have evolved twice under two distinct conditions (Skibiel et al., 2013). The first condition is the extreme separation of whale foraging and calving grounds. For example, mysticetes forage in cool high productivity waters while calving occurs in warm nutrient poor waters (Irvine et al., 2017). The second evolution occurred in phocids where a brief and highly synchronous lactation period is advantageous due to dependance on ephemeral and relatively unpredictable seasonal ice that supports resting, breeding, parturition, and nursing (Schulz and Bowen, 2004; Skibiel et al., 2013). The relatively unstable nature of the seasonal ice cover likely contributed to the evolution of precocial offspring with rapid growth rates in conjunction with precipitous transfer of energy via nutrient dense milk from dam to offspring (Schulz and Bowen, 2004). Years with poor ice conditions (thin, late onset, or early retreat) are associated with poor pup production in phocids dependent on ice for parturition and lactation (Laidre et al., 2008).

**Figure 2.**
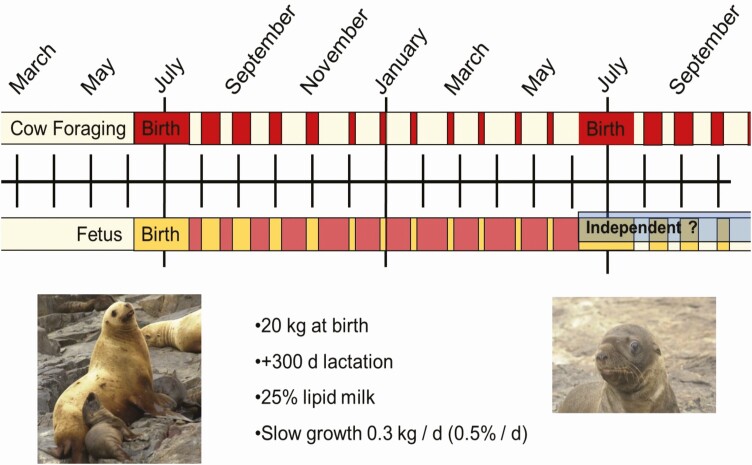
The Steller sea lion (*Eumetopias jubatus*) Income-Foraging lactation strategy diagramed above provides an example of the typical otariid pattern although significant species differences exist in duration of lactation and transition to independent foraging. Top bar indicates female pattern of foraging at sea (light yellow) and fasting on land (red) while provisioning her pup. Bottom bar provides pup pattern of nursing (dark yellow) and fasting (light red) on land while the female is foraging at sea. Pups are nursed consistently for 2 weeks (range from 4 to 14 days) immediately post-partum. After which, pups and females begin an alternate foraging-fasting cycle where pups fast on shore while females forage at sea. Most Steller sea lion pups wean at 12 months of age; however, the transition to independent foraging (blue) is not discrete and pups may continue to nurse well into the juvenile phase at 2 or 3 years of age (Hastings et al., 2021).

Sea lions, fur seals (otariids), and some phocids exhibit an income-foraging lactation strategy (Figure 2; Schulz and Bowen, 2004). Females arrive at rookeries (sites of parturition and nursing) with energy reserves sufficient to nurse pups consistently for a few days up to 2 weeks. Following this discrete nursing period females begin to alternate between foraging at sea while pups are fasting on rookeries, and the dam fasting on land while providing nutrition to the pup (Figure 2). This fasting-foraging cycle continues for months to years dependent on species and population (Hastings et al., 2021), and foraging trip duration increases as the pup ages (Higgins and Gass, 1993; Arnould and Boyd, 1995).

A unique physiological adaptation has occurred in otariids to maintain milk production despite prolonged periods, three weeks or more (>21 days), of foraging at sea with interspersed production of large quantities of energy dense milk on land. In most mammals, mammary gland involution begins quickly following delayed milk removal (Li et al., 1997) and increased hydrostatic pressure cause by milk accumulation downregulates gene expression of milk proteins (Lefèvre et al., 2010). However, in otariids the gene that codes for alpha-lactalbumin, important for lactose synthesis, is knocked down (Reich and Arnould, 2007; Sharp et al., 2008). This is hypothesized to contribute to the reduced water content of milk and also to the lack of involution despite days to weeks of no milk removal while the dam is foraging at sea. In contrast to otariids, mammary evacuation is required to maintain lactation in phocids (Lang et al., 2005). While it is unknown how quickly involution occurs due to increased hydrostatic pressure, lactation interrupted by foraging bouts as short as 4‐6 days in harbor seal dams (*Phoca vitulina,* small body phocid) results in a dramatic reduction of milk fat and increase in protein content indicating involution (Lang et al., 2005).

## Dam/Cow Duration of Lactation

The current 33 species of pinniped exhibit exceptional diversity in length of lactation. Both environment and phylogeny appear to be important in the evolution of lactation strategy and duration unique to each family (Skibiel et al., 2013; Berta et al., 2018). In general, phocids (19 species of true earless seal) utilize the capital-fasting strategy (Figure 1) and nurse pups for days to weeks, with a few exceptions including tropical Mediterranean monk seals (*Monachus monachus*), temperate harbor seals, and Antarctic Weddell seals (*Leptonychotes weddellii*). In contrast, otariids (8 species of fur seal and 5 sea lion species) using the income-foraging strategy where lactation lasts months to years (Figure 2; Schulz and Bowen, 2004). Duration of lactation has significant impact on total maternal energy investment with short lactation investing less energy mostly as fat, and with longer lactation periods, females invest more energy as well as protein (Costa and Maresh, 2017).

Utilizing summary data provided by Schulz and Bowen (2004) to calculate daily energy transfer (milk energy) relative to maternal body mass, phocids (*n* = 3 species) transfer 36‐20 (MJ/kg)/d, with the exception of hooded seals approximately 80 (MJ/kg)/d. The relative energy transfer is impacted both by composition of milk and duration of lactation. Otariids (*n* = 4 species, Schulz and Bowen, 2004) provide significantly less daily energy relative to maternal mass 7‐23 (MJ/kg)/d; however, given this investment continues for months to years the overall energy investment is much higher compared with phocids. Despite numerous studies evaluating the cost of lactation in pinnipeds, daily energetic cost for the female remains a significant bioenergetics question (McHuron et al., 2022, 2023).

**Figure 3.**
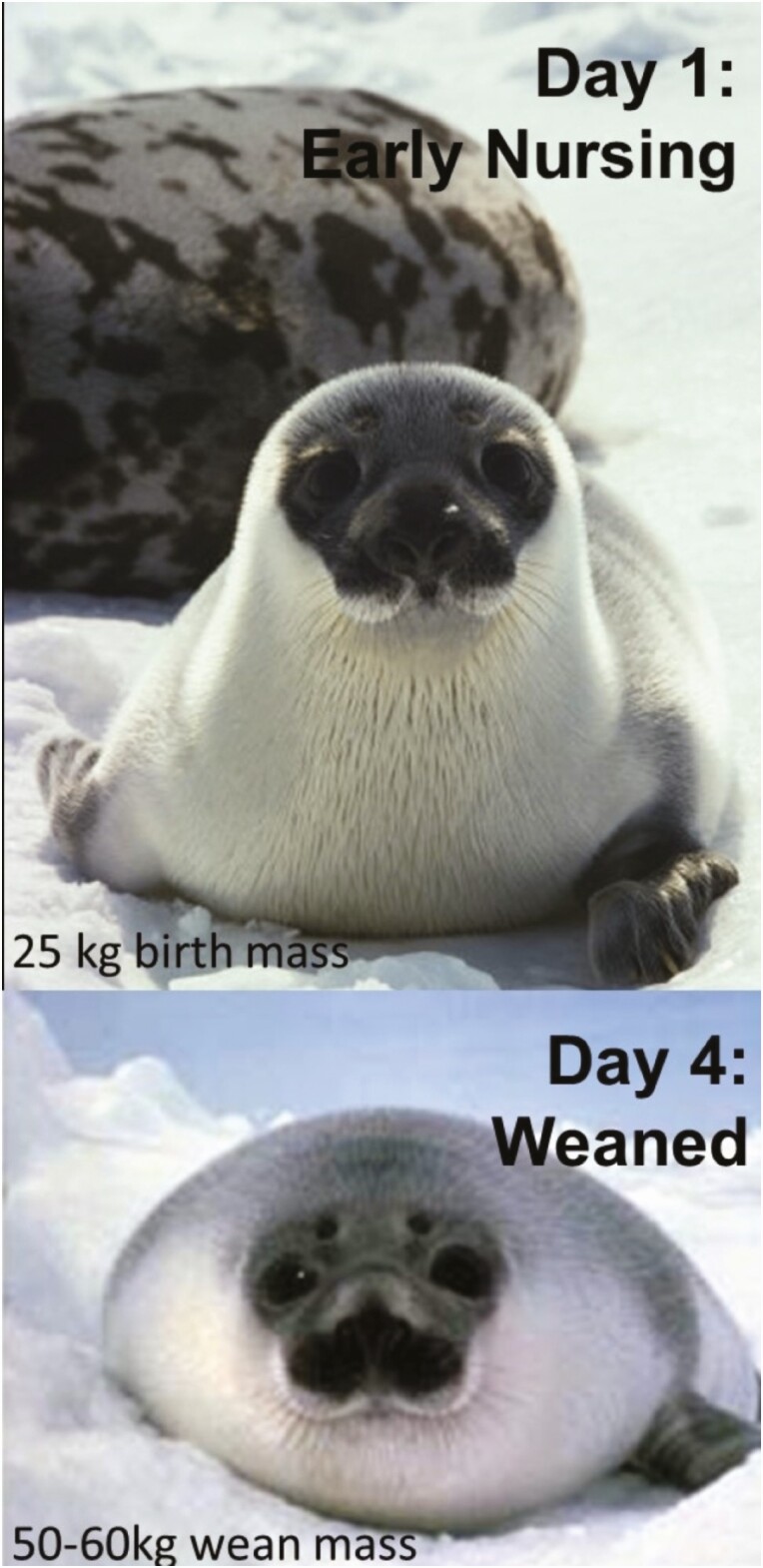
Hooded seal (*Cystophora cristata)* pups consume 10 L·d^−1^ of milk with 60% lipid and 5% protein for 4 days gaining approximately 30% of their birth mass each day (Oftedal et al., 1993; Lydersen et al., 1997). Gross energy content of hooded seal milk is 5.9 kcal/g (Oftedal et al., 1993). Milk energy consumption is 60,000 kcal (251 MJ) per day leading to a 7 kg gain in body mass. This energy consumption equivalent would be similar to a 6-year-old human [average range 16‐27 kg, CDC Growth Chart] consuming 109 McDonald’s Big Mac [550 kcal (2.3 MJ), 30 g fat, 45 g carbohydrate, 35 g protein each] per day for 4 days although the fat percentage would still be less than hooded seal milk.

Most phocids lactate for 2‐4 weeks; ranging from 4 days in the hooded seal (Figure 3), the shortest lactation duration of any mammal (Oftedal et al., 1993), up to 4 months in the cave dwelling Mediterranean monk seal (Aguilar et al., 2007). The capital-fasting strategy requires downregulation of maternal maintenance energy needs to compensate for the increased energy required for lactation, and individual failure to reduce maintenance needs results in early termination of lactation (Shuert et al., 2020).

Energy transfer relative to maternal body mass does appear to influence length of lactation with larger bodied individuals able to transfer a greater quantity of energy in a shorter period of time (Lee et al., 1991; Costa and Maresh, 2017). Within species smaller, younger, or leaner (lower mass to length ratio) individuals have less resources to transfer to offspring (Lee et al., 1991; Costa and Maresh, 2017). Each of these factors has been shown to reduce energy transfer to pups through reduced lactation duration in both phocids and otariids or increased maternal foraging trip duration in otariids (Wheatley et al., 2006; Merrill et al., 2021). Thus, reducing the overall time nursing the pup.

While both otariids and phocids have highly variable lactation duration, otariids have much longer lactation lengths compared with phocids. Fur seals average duration is 4 months while most sea lions nurse for 11 months, except Australian sea lions that nurse for 17 months (Schulz and Bowen, 2004). Steller sea lions (*Eumetopias jubatus*) exhibit the typical income-forging lactation strategy (Figure 2) where females fast while pups nurse during the approximately 2 week (range from 4 to 14 days) immediate post-partum period (Hood and Ono, 1997; Burkanov et al., 2011). Older, larger females tend to have a longer postpartum period before leaving the rookery to forage, leaving the pup to fast until the cow returns (Yamsky et al., 2007). Even within the same species and year, postpartum foraging duration varies by location (Yamsky et al., 2007). While most Steller sea lion pups wean just before parturition of a new pup, it is not unusual to see a 2- or 3-year-old nursing (Hastings et al., 2021).

While twinning is rare (>0.4% of all births) in pinnipeds since most species give birth to one pup per year, twinning has been genetically confirmed in walrus, Weddell seals, southern elephant seals, and Antarctic fur seals (Gelatt et al., 2001; Hoffman and Forcada, 2009). More commonly, observations of females nursing more than one pup is due to fostering (also termed adoptive suckling, allosuckling, or allonursing) by the female or milk-stealing initiated by a nonfilial pup (Sepúlveda and Harcourt, 2021). While true fostering until weaning is uncommon, milk-stealing occurs frequently among both otariids and phocids with obvious nutritional benefits to the nonfilial pup, although very risky due to aggressive retaliation from both the female and conspecific pup (Sepúlveda and Harcourt, 2021). Negative impacts of fostering on the adult female and her pup are observed with extended duration of lactation (Civil et al., 2021). Local bathymetry, prey quality and abundance impact female foraging trip duration for otariids utilizing the income provisioning strategy. For example, in the North Pacific adult female Steller sea lions foraging on low quality prey such as pollock have much longer foraging trip duration compared with females consuming primarily mackerel, a higher quality, energy dense prey item (Sinclair and Zeppelin, 2002; Burkanov et al., 2011). Bathymetry likely influences the distribution and predictability of prey resources while access to high quality, energy dense, prey facilitates a more rapid repletion of energy reserves needed to continue suckling the pup (Burkanov et al., 2011).

Environmental changes resulting in reduced prey quantity or quality negatively impacts female body mass, fat reserves, and percentage of energy expenditure during lactation (Wheatley et al., 2006). These changes are also reflected in poor offspring growth and body condition with decreased mass and fat reserves at weaning. In years with warm ocean temperatures, such as El Niño events in the North Pacific, results in reduced quantity and quality of prey, increased foraging trip duration for females and delayed weaning for pups (Merrill et al., 2021). In Steller sea lions two maternal behavioral factors contribute to reduced survival in El-Niño years (Hastings et al., 2021). First, due to a shorter immediate postpartum period (<1 wk, typically 2 wk, Figure 2) pups initiate the post-partum fast with reduced body fat stores relative to more typical years with two weeks of maternal investment prior to the first postpartum fast (Hood and Ono, 1997). Second, maternal foraging trip durations are longer resulting in longer periods of fasting for the pup (Hood and Ono, 1997; Hastings et al., 2021). Similarly, in the southern oceans significant decline in pup production is associated with increased/high sea surface temperature (SST) in otariid species (Beauplet et al., 2005). Periods of increased SST impact Antarctic fur seal (*Arctocephalus gazella*) reproduction beyond one breeding cycle, likely due to low availability of prey (Forcada et al., 2005). Environmental impacts on the lactation triad have been observed in multiple otariid species including changes in milk composition, decreased nursing bouts, increased foraging time at sea, and/or extended duration of nursing including abandonment of current pup in lieu of continued suckling of older sibling (Higgins and Gass, 1993; Forcada et al., 2005; Trillmich and Wolf, 2008; Hastings et al., 2021). Climate change has the potential to decrease reproductive success both in species dependent on ice for partition and lactation and within more temperate and tropical species due to the challenge of maintaining sufficient energy intake for lactation, reducing pup survival (Kovacs et al., 2012; McHuron et al., 2023).

## Pinniped Milk

### Macronutrient composition

Across all mammals, including eutherian and placental mammals, phylogeny is a better predictor of milk composition and energy density than environmental factors such as latitude and predictability of prey resources (Lefèvre et al., 2010; Skibiel et al., 2013). In contrast, within groups of closely related pinniped species environmental factors are important for determining lactation length which influences composition of milk and rate of energy transfer (Berta et al., 2018).

**Figure 4.**
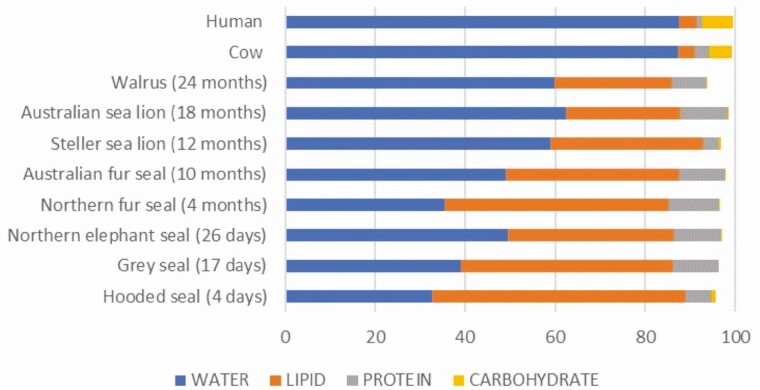
Proximate composition of select pinniped milks (g/100 mL) relative to humans and cows. Pinniped length of lactation provided in parentheses. Example income-foraging strategy otariids listed include two sets of sympatric sea lion-fur seal pairs. Australian sea lion (*Neophoca cinerea*) and fur seal (*Arctocephalus pusillus*) overlap distribution across the southern coastal region of Australia. Steller sea lions (*Eumetopias jubatus*) and Northern fur seals (*Callorhinus ursinus*) inhabit similar coastal regions across the North Pacific Rim of Fire. Three phocids [northern elephant seal (*Mirounga angustirostris*), grey seal (*Halichoerus grypus*), hooded seal (*Cystophora cristata*)] with the capital-fasting lactation strategy typify the extreme low water, high fat, energy dense milk composition of these unique mammals. Carbohydrate in pinniped milk ranges from undetectable to 1%. Data presented are modified from Oftedal et al. (1987), Roy et al. (2020) and McHuron et al. (2023).

Phocid milk matures rapidly from colostrum to mature milk within 24 hours in grey seals (Lowe et al., 2017) and almost immediately following birth in hooded seals (Oftedal et al., 1987). The composition of mature pinniped milk (Figure 4) provides an energy dense, low water, high lipid milk to their offspring (McHuron et al., 2023). Water content of pinniped milk ranges from approximately 35% to 65% compared with cow and human milk that contains approximately > 85% water (McHuron et al., 2023). In contrast, fat content of pinniped milk ranges from 25% to 60% lipid compared with the 4% milk fat in cow or human milk. Pinniped milk is also rich in protein with an average of 11% ranging from 9% to 14%, compared with 1% to 3% in humans and domestic species (Schulz and Bowen, 2004; Roy et al., 2020).

Pinnipeds initiate lactation with colostrum with less fat and greater protein content. Fat percentage of phocid milk is rapidly increased in mature milk to peak lactation values (Oftedal et al., 1987). The exception is hooded seals that initiate lactation with lipid concentrations similar to peak lactation values. In otariids the increase in fat percentage is more gradual. In some species, peak lactation may require 3‐6 months depending on the typical duration of lactation (Costa and Valenzuela-Toro, 2021). Interestingly, in otariids, no relationship has been found between percentage milk fat female mass, or time on shore before milk collection; however, longer foraging trip duration results in greater milk fat composition (Arnould and Boyd, 1995; Drago et al., 2021). The increased energy density of milk compensates for the additional energy costs pups incur due to extended fasting duration such that neither pup growth rate or rate of milk energy delivery is impacted by the time females are away from the pup (Arnould and Boyd, 1995; Drago et al., 2021).

Milk lipid fatty acid (FA) composition is also unique in marine mammals with large quantities of long chain (≥16 C) saturated (SFA), monounsaturated (MUFA), and polyunsaturated fatty acids (PUFA) that reflect their marine diet (Iverson et al., 2004). Pinniped milk FA, in both phocids and otariids, are comprised primarily (50‐60%) of MUFA, with approximately 20% SFA and 20% PUFA (Wheatly et al., 2008; Fowler et al., 2014; Miller et al., 2017). The percent FA profiles of pinniped milk are quite different from human and cattle milk. Human milk contains 3945% SFA, 33‐45% MUFA, and 8‐19% PUFA, while milk from domestic cattle is comprised of 56‐73% SFA, 23‐30% MUFA, and 2‐6% PUFA (Roy et al., 2020). Specific FA are highly represented in pinniped milk across species notably 16:0 (~10%), 18:1n-9 (~20%), 20:5n3 (5‐10%), 22:6n-3 (~10%) (Brown et al., 1999; Fowler et al., 2014; Miller et al., 2017). Two PUFA (20:5n-3 and 22:6n-3) are known to be critical for human brain development (Martinat et al., 2021) and are highly represented in pinniped milk compared with human milk (Fowler et al., 2014; Watson et al., 2021). However, little is known about the role of PUFA in neurodevelopment of pinnipeds (Watson et al., 2021). Studies suggest that 20:5n-3 is selectively mobilized from dam blubber and deposited into milk (Wheatly et al., 2008; Fowler et al., 2014). Furthermore, this specific PUFA is also selectively mobilized during the postweaning fast of northern elephant seal pups (*Mirounga angustirostris*) highlighting its importance in pinniped growth and development (Noren et al., 2013).

In most mammals, carbohydrate has an important role as an energy substrate for growth, composition of mass gain, neurocognition and immune function (Berger et al., 2020). In contrast to other mammalian milk, in many pinniped species lactose is virtually undetectable in milk (Dosako et al., 1983; Oftedal, 1993; Watson et al., 2021). Direct measure of carbohydrate in pinnipeds is important since indirect measures often lead to erroneous results indicating greater concentrations of carbohydrate than are actually present (Oftedal et al., 2014). Decreased or absent lactoses concentrations likely facilitate the reduced water content and greater lipid accumulation in pinniped milk. A mutation in the alpha-lactalbumin is present in several otariid species including California sea lion (*Zalophus californianus*), Cape fur seals (*Arctocephalus pusillus*), Antarctic fur seals, and walrus that prevents lactose production and is thought to allow maintenance of lactation with long (up to 23 days) inter-suckling interval by preventing involution (Reich and Arnould, 2007; Sharp et al., 2008).

While lactose is undetectable in most pinniped milks direct analysis has revealed other carbohydrates of interest that likely have roles beyond basic nutrition source in pinniped milk discussed further in bioactive section below.

### Bioactive compounds

Milk bioactive compounds and related health outcomes are a highly active area of research in human lactation (Bardanzellu et al., 2020; Christian et al., 2021); however, limited research is available for pinnipeds. Bioactive compounds include, but are not limited to, immunoglobins, hormones, proteins, oligosaccharides, white blood cells, peptides, cytokines, chemokines, and micro-RNA (Christian et al., 2021). Oligosaccharides known to contribute to the gut microbiome, such as fucosyllactose and sialyllactose, are found in phocid milk immediately post birth but decline rapidly (Lowe et al., 2017; Watson et al., 2021). In addition, myoinositol is present at 123 mg/100 mL in northern fur seal (*Callorhinus ursinus*) milk (Dosako et al., 1983). These concentrations far exceed values in cow and rodent milk (4 and 77 mg/100 mL, respectively; Byun and Jenness, 1982). In human infants, serum myoinositol concentrations are greater in neonates than adults and increase with human milk feeding although the role in neonatal physiology is unknown (Pereira et al., 1990). Myoinositol has been extensively studied in humans as a preventative treatment for gestational diabetes in overweight and obese humans (Mashayekh-Amiri et al., 2022). The role of this carbohydrate in naturally obese species such as pinnipeds is unknown.

Metabolic hormones such as insulin-like growth factor (IGF)-I and ghrelin in pinniped milk likely contributes to gut maturation, rapid adipose accumulation and sustained appetite while consuming high volumes of energy dense milk and during other hyperphagic states (Dailey et al., 2016; Avery, 2017). In humans, greater concentrations of IGF-I in milk are associated with greater mass gain at 1 year old, but decreased body mass at 3 and 5 years of age (Galante et al., 2020). Limited research is available for IGF-I concentrations in seal milk; however, concentrations are greater in phocid milk compared with human milk (Avery, 2015). Circulating blood concentrations of IGF-I in phocid pups is positively associated with growth rate and adipose accumulation (Daily et al., 2016, 2020). Concentrations of ghrelin in both seal milk (exogenous) and circulating (endogenous) concentrations in pups suggests that ghrelin is increased during life history events that require hyperphagia independent of gut fill (Avery, 2015; Dailey et al., 2016). While ghrelin typically increases in response to gastric emptying to stimulate intake, during these periods of hyperphagia the opposite pattern is observed. Increased circulating ghrelin is observed in pinniped serum, during compensatory growth following fasting, and in hyperphagic periods associated with seasonal fattening (Dailey et al., 2016). Role of metabolic hormones and other bioactive compounds in pinniped milk remains another under-explored area of research.

## Pup Mass and Growth

While terrestrial mammals give birth to relatively small, altricial offspring (~1‐10% of maternal mass at birth), marine mammals birth precocial young that are much larger relative to maternal mass (~2‐20%; Costa and Maresh, 2017). This larger relative fetal growth rate requires significant maternal energy investment during gestation as well as vitamins, minerals and macronutrients essential for normal development and growth; however, this greater gestational investment results in a larger, more developed offspring with significant locomotor capacity almost immediately after birth (Costa and Merash, 2017).

Postnatal growth during the nursing period is a significant predictor of first year survival in both otariids (Beauplet et al., 2005; Hastings et al., 2021) and phocids (Muelbert et al., 2003; Bowen et al., 2015). In phocids, elevated lipoprotein lipase activity in pup plasma and adipose facilitate the rapid and efficient transfer of milk lipid directly deposited to pup blubber stores (Iverson et al., 1995; Mellish et al., 1999; Crocker et al., 2001). Surprisingly, percentage milk fat is not associated with either absolute growth rate (kg/d) or relative to birth mass [(kg/d)/kg] (Schulz and Bowen, 2004). Since growth rate relative to pup birth mass [(kg/d)/kg] is related to rate of energy transfer (kJ/d), seals that maximize energy transfer rates (fat, protein, volume) have reduced period of lactation (Costa and Maresh, 2017).

### Growth strategies

In sexually dimorphic otariids, differential growth patterns are observed between male and female pups related to maternal provisioning (suckling bout duration, milk energy density, duration of lactation) and food availability (environment quality). In general, females receive greater fat milk that is not limited by maternal resources or food availability, while males receive reduced fat milk and provisioning may be limited when the cow has reduced energy reserves (Georges and Guinet, 2000; Drago et al., 2021). Research in other pinniped species have found both no differences in provisioning between sexes and some difference that are hypothesized to relate to pup mass, not sex (Salogni et al., 2018; McHuron et al., 2023). Studies in other mammals have mixed results with a general trend toward greater energy and volume provided to males; however, this varies by milk constituent, energy, protein, or volume (Lee, 1996; Galante et al., 2018). Additional research investigating total available milk energy including energy density and milk yield need to be quantified together to determine what drives sex differences in milk constituents and if differences in maternal provisioning exist. Sex differences in endogenous metabolic hormones such as growth hormone, ghrelin, and IGF-I that impact differential nutrient utilization and growth rate of males and females has been well studied in terrestrial species; however, limited studies have been conducted in pinnipeds (Lee, 1996; Richmond, 2008). In addition to sex differences in maternal contribution of quantity, composition, and volume of milk, pups may also differ in the utilization and allocation of these nutrients to impact differential growth rate between sexes.

Phocid growth rate can vary from 7 kg/d (30% of birth mass) in hooded seals, to 2 kg/d (7% of birth mass) in Weddell seal, and with smaller phocids, such as harbor seals, gaining only 0.6 kg/d (5% of birth mass; Schulz and Bowen, 2004). In phocids utilizing the capital-fasting lactation strategy, pup growth (fast-capital growth strategy) is primarily lipid accumulation as specialized subcutaneous adipose tissue termed blubber. In contrast, income-foraging otariids growth rate is much slower around 1 kg/d (0.9‐1.5% of birth mass), termed slow-income growth strategy (Schulz and Bowen, 2004). Furthermore, species differences in growth strategies are apparent. In two closely related fur seals, Antarctic fur seals have faster lean growth and physiological development and energetically intensive behavior that result in early development of foraging skills and younger weaning age compared with sympatric subantarctic fur seals (Arnould et al., 2003).

Despite large variation in growth rate (Figures 1 and 2), there appears to be a threshold value where offspring attain approximately four-times birth weight before weaning (cessation of milk consumption, independent of supplementary food intake; Lee et al., 1991). This has been observed not only for pinnipeds but also across mammalian taxa (Lee, 1996). This suggests that individual growth trajectories (growth rate) determine the length of lactation and timing of weaning.

## Response to Environmental Change

Unsurprisingly, climate change has profound effects in polar regions with loss of ice for pagophillic (ice-loving species) impacting reproductive success and population trends (Kovacs et al., 2012). However, subpolar, temperate, and even tropical marine mammal species are also negatively impacted by climate change (Gulland et al., 2022). Studies highlighted throughout this review identified varied and profound effects of increase intensity and frequency of warm ocean events on the pinniped lactation triad regardless of latitude. Warm ocean events reduces food quality and quantity impacting maternal forging success and energy reserves prior to partition documented in both phocids and otariids (Blondin et al., 2022). Maternal body condition determines the energy allocated to offspring, offspring growth and development, and hence survival (Kovacs et al., 2012; Blondin et al., 2022; Gulland et al., 2022). The impact of environmental variability on the pinniped lactation triad is well documented with alterations to maternal provisioning strategy, energy allocation to pups, pup growth rate, and survival all negatively impacting population trends well beyond one year.

## Conclusion

The pinniped lactation triad offers a unique opportunity to investigate exceptional diversity in lactation strategies including varied lactation duration, milk composition including metabolic hormones and other bioactive compounds, provisioning patterns, and resulting pup growth. Pinniped lactation adaptations, including high fat, energy dense milk, delivered rapidly to precocial offspring resulting in precipitous offspring growth, may illuminate new areas of research for exploration in both human and domestic animal lactation.

## About the Authors

**Dr. Julie Avery** is a research assistant professor at the Water and Environmental Research Center at the University of Alaska Fairbanks, Dr. Julie Avery examines lactation and growth from a Conservation Physiology perspective. She holds a PhD in Animal Science and is also a Registered Dietitian and Certified Lactation Counselor She has been studying pinniped perinatal physiology for more than 20 years and has participated in pinniped research expeditions in Alaska, Russia, and Antarctica. Her current research is supported by the National Science Foundation, Office of Polar Programs.

**Dr. Steven Zinn** is a professor of Animal Science at the University of Connecticut. He joined the faculty at the University of Connecticut in 1990 after earning his B.S. degree from Cornell University and his M.S. and Ph.D. degrees in the laboratory of H. Allen Tucker at Michigan State University. His research is focused on growth physiology and endocrinology in domestic species with an emphasis on maternal nutrition. From 2008 to 2013, Dr. Zinn was Editor-in-Chief of the Journal of Animal Science and is a founding member and the inaugural Editor-in-Chief of Animal Frontiers from 2011 to 2013. He served as President of ASAS from 2017 to 2018, was named an ASAS Fellow in 2015 and received the ASAS Morrison Award in 2021.
